# The Potential Role of Nonhuman Primate Models to Better Comprehend Early Life Immunity and Maternal Antibody Transfer

**DOI:** 10.3390/vaccines9040306

**Published:** 2021-03-24

**Authors:** Julie Sartoretti, Christiane S. Eberhardt

**Affiliations:** 1 Center for Vaccinology, Department of Pathology and Immunology, Faculty of Medicine, University of Geneva, 1211 Geneva 4, Switzerland; julie.sartoretti@unige.ch; 2 Department of Woman, Child and Adolescent Medicine, Geneva University Hospitals and Faculty of Medicine, 6 rue Willy-Donze, 1211 Geneve 4, Switzerland; 3 Center for Vaccinology, University Hospitals of Geneva, 1205 Geneva, Switzerland; 4 Emory Vaccine Center, Emory University, Atlanta, GA 30322, USA

**Keywords:** early human life immunity, vaccinology, nonhuman primate model

## Abstract

Early life immunity is a complex field of research and there are still gaps in knowledge regarding the detailed mechanism of maternal antibody transfer, the impact of maternal antibodies on infant vaccine responses and the ontogeny of human early life immunity. A comprehensive understanding is necessary to identify requirements for early life vaccines and to improve early childhood immunization. New immunological methods have facilitated performing research in the youngest, however, some questions can only be addressed in animal models. To date, mostly murine models are used to study neonatal and infant immunity since they are well-described, easy to use and cost effective. Given their limitations especially in the transfer biology of maternal antibodies and the lack of infectivity of numerous human pathogens, this opinion piece discusses the potential and prerequisites of the nonhuman primate model in studying early life immunity and maternal antibody transfer.

## 1. Introduction

The ontogeny of early life immunity is a complex field of research, partly due to the vulnerable population of interest. In addition, the infant immune system has distinct features compared to adults, responding to the exclusive conditions during this initial phase of life. Being born from a mostly sterile environment [1], the newborn lacks specific immune memory against aggressive microbes and is only passively protected through transferred maternal antibodies. While the immune system needs to develop a certain tolerance against commensal microorganisms, it is required to balance it against an effective immune response to pathogens. These diametrical requirements explain the vulnerability of the youngest facing infectious diseases. Neonates are at risk of developing fulminant severe infections against most pathogens, and infants tend to have more severe viral infections and an increased susceptibility to various bacteria, for example encapsulated germs, and in particular *Streptococcus pneumoniae* [2]. In 2018, the mortality rate for children under the age of five years was 3.9%, and approximately half of it was due to communicable diseases, which translated to 2.5 million young children dying that year of potentially vaccine-preventable diseases [3]. This shows the need for a better understanding of early life immunity which could help improving immunization, thus saving lives. Nevertheless, several research hypotheses are difficult to investigate in human newborns calling for the optimal surrogate animal model.

This opinion piece briefly summarizes the state of knowledge of early life immunity and of maternal antibody transfer, discusses currently used animal models and elaborates the potential role of nonhuman primates (NHP) in addressing open research questions.

### 1.1. Early Life Immune Responses

Ontogeny of early life immunity describes the development of immune responses in newborns and infants usually up to the age of 2 years, and differences to the adult innate and adaptive immune system have been reviewed in detail elsewhere [2,4]. Briefly, studies on whole blood and innate blood cell subsets have shown that the initial innate cytokine signature which ultimately shapes the adaptive immune response evolves with age. The anti-inflammatory state observed in preterm neonates and at birth can be explained as adaptive mechanism to prevent abortion during pregnancy and to facilitate the materno-fetal coexistence [5]. In vitro Toll-like receptor stimulation of whole blood and innate blood cell subsets elicits at birth an anti-inflammatory environment and cytokines promoting T_H_17 T cells, followed after the first weeks of life by a capability to induce antiviral responses. While there is an increase in proinflammatory cytokines over the first months of life and T_H_17 T cell polarizing cytokines decrease, the T_H_1 T cell supporting environment only reaches adult levels at around 2 years of life (reviewed in [2]). Innate cells such as dendritic cells and neutrophils have quantitative and functional differences compared to adult cells, showing for example decreased capacity to present antigens or to respond to stimulation [4,6].

These regulated innate responses elicit early life CD4 T cells that are characterized by a marked polarization toward T_H_2 and T_H_17 phenotypes [7,8]. Subsequently reduced T_H_1 responses are associated with a higher risk of intracellular infections [4] and diminished responses to immunization [9]. Additionally, compromised T cell functions are not linked to interference with maternal antibodies but rather result from suboptimal interactions with antigen-presenting cells [10,11], explaining a decreased B cell activation and response. Impaired germinal center formation is thought to be caused by a delayed development of follicular dendritic cells [12], subsequent decreased T follicular helper responses [13,14] and a low germinal center output of differentiated memory B cells and antibody-secreting plasma cells [8]. Hence the relative dominance of extrafollicular B cell responses eliciting short-lived plasma cells which explain the observed low affinity antibodies that rapidly wane [10]. It has also been shown that the survival of plasma cells in the bone marrow is shortened in early life [15]. This translates clinically into diminished antibody responses and the need for multiple booster immunizations during the first year of life. Additionally, vaccination protocols tend to be less successful when starting too early after birth with short intervals between doses [10]. Even though hepatitis B vaccine, which is given at birth, has been often cited as counter-example, seroconversion after the birth dose can be as low as 10% [16] and booster immunizations are needed to confer optimal protection [17].

The ontogeny of the immune system in early life with distinct features depending on the different developmental phases shows the need for neonatal vaccine formulations that fulfill the complex requirements. New immunological research techniques allow to generate reliable data with less than 10 mL of blood [4], enabling sufficient sample collections on newborns and help broaden research possibilities with human samples. However, there is still a need for adequate animal models such as to test new vaccine formulations and adjuvants or to perform challenge experiments.

#### 1.1.1. Animal Models Reflecting Human Early Life Responses

Animal models developed to better characterize immune responses in early life have to fulfill several criteria such as analogy to the human immune system, feasibility and cost-effectiveness. The greatest challenge is that the ontogeny of immune responses of chosen animals need to be as close as possible to humans and that the timing of different developmental stages in the animal compared to humans need to be meticulously defined.

Especially in terms of feasibility and cost-effectiveness, it is obvious that the murine model is an unmissable option and has been the main animal model used in pediatric vaccinology and immunology to date. Mice have similar early life immune patterns as infants: they show similar distinct innate responses, elicit limited Th1 (IFN-γ), increased T_H_2 responses [18], and lower CD8^+^ T cell responses [10]. It has been shown that early B cell activation is limited, germinal center responses are impaired [19] and that antibodies rapidly wane after vaccination [10,19], reflecting a reduced plasma cell persistence in the bone marrow [20]. Even though immune read-outs detectable in the peripheral blood seem identical between human infants and mice, it is still unclear if the underlying mechanisms are similar between both species [20]. Usually 1-week-old pups are used as neonatal model, and mice aged 2–3 weeks for infant models. Different strains of laboratory mice also elicit distinct immune responses. For example, Balb/c mice tend to skew T cell responses toward T_H_2 phenotypes and often elicit higher antibody titers, whereas C57BL/6 mice polarize toward T_H_1 and cellular immune responses [21], indicating the necessity to wisely choose the optimal mouse strain for each experiment. Limitations of the murine model include their short lifespan which can impede investigations such as long-term effects of vaccination. Most importantly, human infectious agents are not always infectious or symptomatic in mice, which renders this animal model inadequate for challenge experiments for those diseases. Human influenza virus does not naturally infect mice [22] and virus adaptations had to be made for murine influenza models, or different bacterial strains need to be used, as done for *Staphylococcus aureus* to mimic human intraperitoneal infection [22]. In order to overcome these challenges, humanized mouse models have been engineered, for example through human hematopoietic stem cell transfer to immunodeficient neonatal mice. More than 4 weeks after transfer, these mice were shown to develop functional adaptive immune responses, in particular regarding B, T and dendritic cells, and susceptibility to some human viral infections such as Epstein–Barr virus [23] (reviewed in detail in [24]). Despite those constraints, murine models are relatively inexpensive and easy to use and thus are model of choice when suitable in responding to specific early life immunity questions.

#### 1.1.2. What Nonhuman Primate Models Can Add

NHPs have unmatched genetical, physiological and behavioral resemblances with humans [25] and Chimpanzees (*Pan troglodytes*) are the most closely related. However, they are rarely used because of their endangered status, availability and cost. Hence less related monkeys, called Old World Monkeys, are currently employed, such as African green monkeys (*Chlorocebus aethiops*), rhesus monkeys (*Macaca mulatta*) and other types of macaques [21].

Some studies necessitate animal models that can mimic the pathophysiology of infections found in humans. Compared to murine models, there are multiple pathogens that affect NHPs and humans in the same way, such as Zika, Ebola, dengue [26] and chikungunya [27] viruses and *Mycobacterium tuberculosis* [28], whereas African green monkeys and macaques are not susceptible to human metapneumovirus [29]. Of special interest for early life immunity is the respiratory syncytial virus (RSV), for which symptoms and age-dependent severity are similar in both species [30]. NHPs have also been used as model in the field of vaccinology to compare the efficacy of whole-cell pertussis vaccine with the acellular vaccine in infant baboons. Wolfe and colleagues chose NHPs as they were the animal model closest related to humans [31] with similar symptomatology and contamination route [32]. The use of NHPs allowed to compare pertussis infection in naïve animals, animals vaccinated with acellular pertussis vaccine or whole-cell vaccine and convalescent animals. Results confirmed a finding already suspected in humans: acellular pertussis vaccine is less efficient in preventing infection and further transmission compared to the whole-cell pertussis vaccine [32]. Nevertheless, the NHP model is very complex given a variety of NHP species that have distinct features. It also has several practical limitations: the total number of animals per study and the number of samples taken per animal are strictly limited by law. It remains a costly model which needs special care facilities and training [4]. Immunological reagents validated for NHP can be scarce [32], but might often be replaced by those used for humans as they can be cross-reacting [26].

However, a fundamental question is the adequacy of the infant NHP model for human infants. The similarities in immune age-related changes between NHPs and humans are important to decipher before employing NHPs in the assessment of early life immunity. We know that NHPs have a similar immunosenescence as humans [25]. In terms of early life immunity, there are only few studies that show a similar development of the immune system during fetal life compared to humans [33], but there are many unknowns regarding the early life NHP immune system.

#### 1.1.3. Next Steps to the Use of NHPs in Assessing Early Life Responses

Thorough studies are needed to determine if NHPs are an adequate model to parallel the ontogeny in humans. We first need to fully comprehend the developmental stages of the immune system and their timing, to then determine what age in NHPs correspond to human neonates or infants. For mice, 1-week-old pups are used as model for human neonates, whereas mostly 3-weeks-old mice are used as surrogate for infants. We suggest performing for example a vaccine study characterizing in detail the immune response to routine childhood immunization in NHPs and to compare these findings to humans. If analogies are confirmed, NHP could be a promising model to use in challenge experiments and in the assessment of reactogenicity and immunogenicity of novel adjuvants that could help improve early life immunization.

Another potential advantage of infant NHPs would be the assessment of mucosal immune responses to vaccination similar to adult models for RSV and SARS-CoV-2 [34,35]. Local vaccines for strictly mucosal pathogens aiming to boost IgA levels are currently studied, for example nasal *Bordetella pertussis* vaccination [36], or are already employed such as rotavirus vaccines. NHPs could help to better assess local immune responses, to understand mechanisms of asymptomatic colonization and to test efficacy of new candidates in challenge experiments. However, prerequisite is a careful validation of the NHP model [37,38].

### 1.2. Transfer of Maternal Antibodies

Passively transferred maternal antibodies provide initial protection to the newborn at birth. Maternal IgG antibodies are mostly transferred through the placenta while IgA are transmitted via breastfeeding and are mainly protecting the mucosal lining of the infant. Maternal antibodies are known to progressively wane after birth, creating a window of vulnerability in the first months of life until infants elicit their own active immunity through vaccination and exposure. There are two approaches to shorten this vulnerable period: early life immunization and maternal vaccination with subsequent transplacental transfer of maternal antibodies. With the introduction of pertussis vaccination during pregnancy less than a decade ago, there is increasing research interest in the optimal timing of maternal immunization, the mechanisms of transplacental transfer and the influence of maternal antibodies on infant vaccination. Nevertheless, maternal vaccination has been recommended by the WHO since the 1960s for tetanus [39,40], an intervention that has greatly decreased neonatal mortality and morbidity [40]. The first studies date from the same period when Gitlin and colleagues injected pregnant women with radio-iodinated γ_2_-globulin and labeled albumin. They demonstrated at birth that mainly IgG was transferred in a time-dependent manner to the newborn, reaching a maternal/infant ratio of 1 at around 3 weeks after injection [41]. The mechanism was later explained by the neonatal Fc-receptors (FcRn) which help transfer albumin and IgG through the placental membrane to the fetus [5]. Interestingly, this receptor can be saturated [5] as seen in lower antibody transfer ratios in mothers with higher antibody titers [42]. It was also revealed that IgG subclasses do not interact similarly with the neonatal Fc receptor at the placental membrane. Indeed, there is a greater transfer of IgG_1_ compared to IgG_2_, while IgG_4_ and IgG_3_ have intermediate affinities to the FcRn [43]. Additionally, there is an impact of the Fc glycosylation patterns on antibody transfer rates, with a preferential transplacental transfer of galactosylated IgG_1_ antibodies [43]. The glycosylation pattern seems to depend on the timing after infection or vaccination and might differ between antibodies elicited following primary and secondary responses [44], and also changes with gestational age at birth [45]. These might be some underlying mechanisms for the observation that antibodies with different antigen specificities tend to be transferred at different rates, as it has been described for antibodies against influenza, tetanus toxoid, herpes simplex virus, streptolysin O, and *Streptococcus pneumoniae* [5].

Regarding the kinetics of antibody transfer, a metanalysis of 540 fetal IgG measurements revealed that antibodies are detectable at 17 weeks of gestational age (GA) and that they increase over time. In 258 paired measurements it was shown that a maternal/fetal ratio of 1 was reached at around 35 weeks of GA [46]. Palmeira and colleagues could detect antibody transfer as early as 13 weeks of GA, but showed a steady increase from 17 weeks of GA onwards [5]. The cumulative transfer of maternal antibodies over time is also underlined by the lower antibody titers found in preterm compared to term infants [47,48]. Parity, maternal age, type of delivery, and birthweight do not seem to have an influence on antibody transfer [49]. When recommending the optimal timing of maternal vaccination, it is important to understand the transfer kinetics. We could show that offspring from mothers immunized during the 2nd trimester (13–25 weeks of GA) rather than during the 3rd trimester (>25 weeks of GA) had higher antibodies titers [50], and that early vaccination also benefited preterm neonates, even if the final titers of transferred antibodies were lower compared to term neonates [50,51].

One of the open questions related to antibody transfer is the interaction of maternal antibodies with future childhood vaccination and a potential blunting of responses. Offspring of mothers immunized with tetanus-diphtheria-pertussis vaccine (Tdap) during pregnancy elicited lower antibody responses to pertussis toxin, even after the booster dose given at 11 months [52]. There are various hypotheses regarding the mechanism of interaction and influence of maternal antibodies on infant vaccine responses and are reviewed elsewhere [53]. Recently, it has been shown in a murine model that humoral responses to adjuvanted influenza vaccination were decreased in presence of maternal antibodies. However, higher maternal antibody titers at vaccination did not prevent B cell activation nor formation of germinal centers. Yet, they affected differentiation of germinal center B cells into plasma cells and memory B cells in a titer-dependent manner and influenced the B cell receptor repertoire [11]. This indicates that maternal antibodies can interfere with infant B cells both in a quantitative and qualitative manner thus shaping infant’s immune responses. However, even if interactions do occur, maternal immunization was found beneficial for both children and mother [54] and the clinical implication of decreased antibody responses has not been elucidated.

There is a need to understand ways of optimizing maternal antibody transfer without compromising future vaccination in the child, for example through a vaccine design or adjuvantation that enhances maternal IgG_1_ and IgG_3_ antibodies or IgG_1_ galactosylation, or through a better understanding of the timing of immunization. More research is needed to decipher the influence of maternal antibodies on the vaccine response in the offspring and on the elicited B cell repertoire. Especially the clinical relevance of blunted vaccine responses needs to be defined which could call for an adaptation of the infant’s vaccination schedule if the mother was immunized during pregnancy.

#### 1.2.1. Animal Models to Study Kinetics and Quality of Maternal Antibody Transfer

For research on maternal antibodies transfer, it is important to understand that humans have a hemomonochorial placenta with an active endosomal antibody transfer, mostly mediated by the neonatal Fc receptor localized on placental syncytiotrophoblasts [55,56], characteristics which greatly differ from most mammalian placentas. In mice, the placental membrane is hemotrichorial and maternal antibodies are mainly transferred by breastmilk [57]. Humanized FcRn mice, which lack mouse FcRn, were shown to transfer IgG transplacentally. However, the level of transferred antibodies was lower compared to titers achieved through subsequent nursing and gastrointestinal transfer, which is opposing to the modalities and kinetics of antibody transfer in humans [58]. Rabbits have a hemodichorial placental membrane, with the same limitations as mice. Sheep do have a hemomonochorial placenta but lack the human ability of transplacental protein transfer [59]. Guinea pigs also have a hemomonochorial placenta similar to humans but differ in the possession of a subplacenta [59]. Interestingly, they do express FcRn, not on the placenta but in their inverted yolk sac [60,61] and IgG transfer during the end of pregnancy has similar kinetics as in humans [61,62]. Despite somewhat comparable mechanisms, antibody transfer in guinea pigs has been proven less efficient than in humans, limiting its use as a model [60]. A summary of the different animal models with their advantages and limitations can be found in Table 1.

#### 1.2.2. Role of NHP in Studying Maternal Antibody Transfer

Old World monkeys, especially baboons and macaques, as well as great apes, have a placenta closest to humans, with a hemomonochorial membrane and similar placental formation [59]. Those primate species have corresponding transplacental antibody transfer mechanisms and express FcRn on their placental membrane cells [60]. There are few studies aiming to understand mechanisms of maternal antibody transfer in NHPs. In rhesus monkeys, IgG was shown to particularly increase during the last two weeks of pregnancy [64] and the same phenomenon was observed during the four last weeks of pregnancy in cynomolgus monkeys [65]. Between species, antibody transfer was variable, with higher antibody transfer in apes and Old World monkeys rather than New World monkeys, but was highest in humans, as the active transfer allowed for higher antibody titers in infants compared to their mothers [66]. This mechanism seems to lack in NHPs as they rely less on the passive protection after birth, and thus remain an incomplete model for maternal antibody transfer [66].

Besides, there is easy access to human umbilical cord blood samples and placenta to elucidate maternal antibody transfer [57] and to answer questions about the immune system at the time of birth. However, its composition is not identical to capillary blood samples of neonates, and thus should be interpreted with caution [67].

#### 1.2.3. Next Steps to the Use of NHPs in Assessing Transfer and Influence of Maternal Antibodies

A NHP model could be used to identify means to improve maternal antibody transfer, assess in-depth the influence of maternal antibodies on the infant immune response to subsequent vaccination and the elicited B cell repertoire. Especially, information on protection against challenge as indicator for clinical relevance of described blunting in the vaccine response would be a valuable exploration. However, results from human studies show increasingly complex details of IgG-subclass and glycosylation-dependent transplacental transfer of antibodies. These findings already discovered in humans would need to be confirmed in NHPs before this model could be used to study materno-fetal antibody transfer and blunting of infants’ immune responses.

## 2. Conclusions

There are gaps in knowledge regarding the ontogeny of early life immunity and the mechanisms and effects of maternal antibody transfer. Animal research models are needed to help find improved early life vaccine formulations that fulfill distinct immunological requirements and confer protection. Mouse models are widely used, however, have limitations in the non-infectivity of human pathogens and are not suited to study transplacental antibody transfer. NHP could be a promising model to study the immune response to vaccination in early life, especially regarding the clinical relevance of blunting through maternal antibodies, to assess mucosal immune responses to vaccination and to improve neonatal vaccine formulations. However, it is to date unknown if the ontogeny of early life immunity in nonhuman primates compares to humans with their distinct developmental stages. This calls for research to better understand the similitudes of early life immunity in humans and NHPs. We suggest conducting in-depth immunological studies with conventional childhood vaccines to prove the accuracy of an early life NHP model.

## Figures and Tables

**Table 1:** Benefits and limitations of each model for early life immunity and maternal antibody transfer studies.

Model	Human-Like Early Life Immunity	Type of Placenta (No of Layers)	Human-Like FcRn	Pros	Cons	Potential Use
Human		Hemochorial (1)	Yes		Limited samples Not useable for vaccine and adjuvant development and mechanistic in vivo studies	TAT ELI
Mouse Conventional Strains * Engineered °	Yes, but corrected for age (neonate = 1 weeks old pups, vs. infant = 2–3 weeks old pups)	Hemochorial (3)	No (°)	-Well-established -Cost-effective compared to large animals	-Breeding facility needed to study specific mouse crosses -Not adequate for assessing antibody transfer at birth as transfer through breastmilk (peak only after around 2 weeks) -By respecting an interval of 3 weeks for vaccine booster doses, mice are already adult -Short life span	ELI
Rabbit/Sheep	NA	Hemochorial (2: rabbit) (1: sheep)	No		Inadequate model for early life and antibody transfer	None
Guinea pig	NA	Hemochorial (1, with a subplacenta)	Yes	Cost-effective	Guinea pig FcRn transfer is less efficient than human FcRn	TAT (?)
Piglets	Shown for example in BCG vaccination [63]	Epitheliochorial	No	Similar responses as humans to some pathogens	Large animal-model	ELI
NHP (Baboons, macaques)	Unknown yet	Hemochorial (1)	Yes	Same response as humans to many pathogens and vaccines	Costly, special care facilities needed and trained personal Similarity in early life immunity needs to be proven before its use	TAT ELI (?)

ELI = early life immunity, TAT = Transplacental antibody transfer; (?) indicates a theoretical potential use that needs to be confirmed through comparative experiments, NA = no data available or not discussed. * C57BL/6 mice elicit mostly a T_H_1 and cellular immune response, whereas Balb/c mice elicit responses that are skewed towards T_H_2 and antibodies. The crossing of male C57BL/6 mice and female Balb/c mice (CB6F1) is a model used for the transfer of (high titers of) maternal antibodies to the pups and to subsequently assess the influence of maternal antibodies on the infant vaccine response [11]. ° engineered mouse strains: similarities to humans in the assessment of ELI depend on mouse strains but need to be corrected for age (neonate vs. infant). For the transplacental IgG transfer in humanized FcRn mice it needs to be shown that antibody titers in the offspring are higher than in the mother as an indicator for sufficient similarities to humans.
